# Proteogenomics analysis reveals specific genomic orientations of distal regulatory regions composed by non-canonical histone variants

**DOI:** 10.1186/s13072-015-0005-9

**Published:** 2015-04-10

**Authors:** Kyoung-Jae Won, Inchan Choi, Gary LeRoy, Barry M Zee, Simone Sidoli, Michelle Gonzales-Cope, Benjamin A Garcia

**Affiliations:** The Institute for Diabetes, Obesity, and Metabolism, Philadelphia, PA 19104 USA; Department of Genetics, Perelman School of Medicine, University of Pennsylvania, Philadelphia, PA 19104 USA; Department of Molecular Biology, Princeton University, Princeton, NJ 08544 USA; Epigenetics Program, Department of Biochemistry and Biophysics, Perelman School of Medicine, University of Pennsylvania, Philadelphia, PA 19104 USA; Department of Agricultural Biotechnology, National Academy of Agricultural Science, 370 Nongsaengmyeong-ro, Wansan-gu, Jeonju-si, Jeollabuk-do 560-500 South Korea

## Abstract

**Background:**

Histone variants play further important roles in DNA packaging and controlling gene expression. However, our understanding about their composition and their functions is limited.

**Results:**

Integrating proteomic and genomic approaches, we performed a comprehensive analysis of the epigenetic landscapes containing the four histone variants H3.1, H3.3, H2A.Z, and macroH2A. These histones were FLAG-tagged in HeLa cells and purified using chromatin immunoprecipitation (ChIP). By adopting ChIP followed by mass spectrometry (ChIP-MS), we quantified histone post-translational modifications (PTMs) and histone variant nucleosomal ratios in highly purified mononucleosomes. Subsequent ChIP followed by next-generation sequencing (ChIP-seq) was used to map the genome-wide localization of the analyzed histone variants and define their chromatin domains. Finally, we included in our study large datasets contained in the ENCODE database. We newly identified a group of regulatory regions enriched in H3.1 and the histone variant associated with repressive marks macroH2A. Systematic analysis identified both symmetric and asymmetric patterns of histone variant occupancies at intergenic regulatory regions. Strikingly, these directional patterns were associated with RNA polymerase II (PolII). These asymmetric patterns correlated with the enhancer activities measured using global run-on sequencing (GRO-seq) data.

**Conclusions:**

Our studies show that H2A.Z and H3.3 delineate the orientation of transcription at enhancers as observed at promoters. We also showed that enhancers with skewed histone variant patterns well facilitate enhancer activity. Collectively, our study indicates that histone variants are deposited at regulatory regions to assist gene regulation.

**Electronic supplementary material:**

The online version of this article (doi:10.1186/s13072-015-0005-9) contains supplementary material, which is available to authorized users.

## Background

The eukaryotic genome is packaged in the nucleus as chromatin, a dynamic arrangement which serves to compact the DNA. Chromatin structure is highly complex as, while packaged, is accessible for selective gene expression and DNA repair. Moreover, chromatin is highly dynamic during chromosome condensation processes such as mitosis and meiosis [1]. The fundamental unit of chromatin is the nucleosome. Nucleosomes are composed of an octamer of histone proteins comprised of two copies each of H2A, H2B, H3, and H4 [2]. Histone N-terminal tails are exposed outside the nucleosomes, and they are heavily modified by dynamic post-translational modifications (PTMs). The deposition of such PTMs modulates chromatin structure, which directly affects the abovementioned DNA-related events [3,4]. Histone PTMs are also among the major drivers of epigenetic memory, as they can be inherited after cell division [5]. Aberrations in PTM relative abundance have been found in several diseases [6,7], which highlights the direct link between histone marks and cell phenotype.

In addition to the canonical histones, there are also protein variants encoded by separate genes [8]. These variants play further important roles in DNA packaging and controlling gene expression [9]. For instance, histone H2A.Z replaces canonical H2A at some 5′ end of both active and inactive genes [10-15]. Recent studies also identified that H2A.Z is enriched at active enhancers, destabilizing the local nucleosome structure and facilitating nucleosome removal [16,17]. Histone H3.3 is specially enriched at transcriptionally active genes as well as regulatory elements [18-22]. Also, unstable H2A.Z/H3.3 double-variant-containing nucleosomes were reported at active promoters, enhancers, and insulator regions [23]. Another H2A variant, macroH2A, is enriched for the inactive X chromosome, and therefore, it has been mainly associated with heterochromatic regions [24,25]. More recently, macroH2A has also been shown to activate genes, although it was still closely associated with the silencing mark H3K27me3 [26]. Such complex panorama of histone PTMs and variants calls for further studies to more accurately define the combinatorial preferences of histone variants and their function for gene regulation.

**Figure 1:**
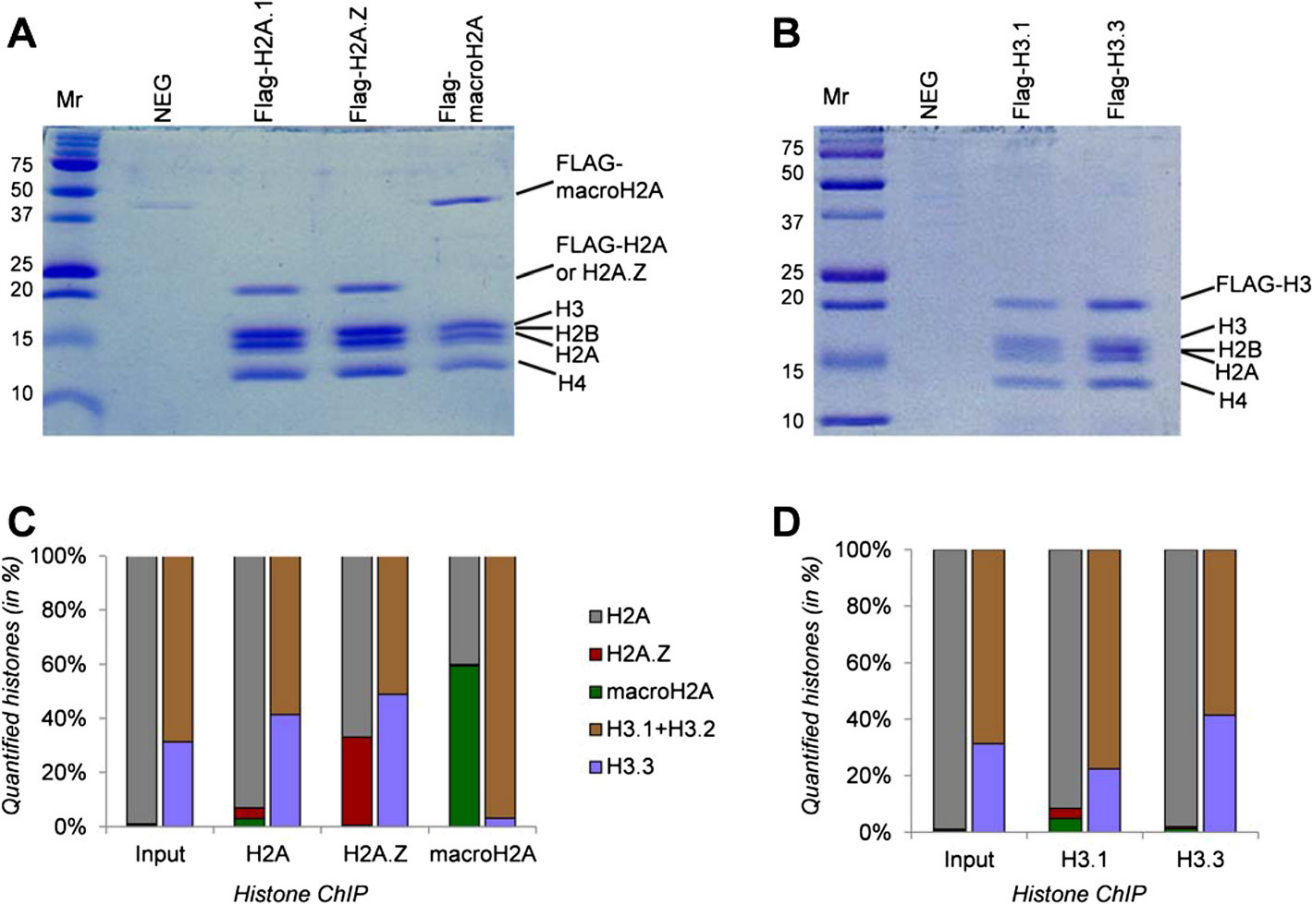
**Analysis of immunoprecipitated mononucleosomes.** Coomassie staining gel representing protein composition of **(A)** FLAG-H2A- and **(B)** FLAG-H3-immunoprecipitated histone samples. NEG represents negative control. **(C)** Relative abundance of canonical histone H2A (gray), H2A.Z (red), macroH2A (green), canonical histone H3 (H3.1 + H3.2, brown), and H3.3 (violet) calculated from the ChIP-MS analysis of H2A FLAG-tagged histones and **(D)** H3 FLAG-tagged histones.

In order to understand the strategic deposition of histone proteoforms and their functional roles, we quantitatively investigated using chromatin immunoprecipitation coupled to mass spectrometry (ChIP-MS) the composition at single-nucleosome resolution of histone-variant-containing mononucleosomes. Moreover, such mononucleosomes were genome-wide mapped using chromatin immunoprecipitation followed by next-generation sequencing (ChIP-seq). In these experiments, we employed HeLa cells expressing either FLAG-tagged canonical histone H3, H3.3, canonical histone H2A, and H2A.Z. Moreover, FLAG-affinity-purified mononucleosomes were analyzed by ChIP-MS (Figure 1A, B) to quantitatively determine histone PTM composition [27]. Hence, our proteogenomic approach allowed us to define chromatin domains containing combinations of histone variants. In particular, we mapped and determined the composition of domains enriched in histone H3.1 and macroH2A, which last is known as repressive signature. More importantly, we observed directional profiles of histone variants, where histone H2A.Z occurred ahead of H3.3 in gene enhancers. This directional pattern co-occurred with the enrichment of RNA polymerase II (PolII), suggesting that PolII has orientation at enhancers and histone variants reflect its transcriptional direction.

## Results and discussion

### Determination of the relative abundance of histone variants

Nucleosomes contain two copies of each core histone type (that is, H4, H3, H2A, and H2B). We used quantitative MS to analyze the ratio of the different H3 and H2A variants in the FLAG-purified mononucleosomes. As expected, canonical H2A was found to be the most abundant variant in nucleosomes purified with either FLAG-H2A or FLAG-H3.1 (Figure 1C and Additional file 1: Table S1). H2A.Z was not observed to be enriched in mononucleosomes purified with histone H3.3, even though previous studies indicate that it should be enriched at enhancers of expressed genes [16,17]. We found a portion of H2A.Z co-existing with H3.3, but this portion is very small and likely quite specialized. Canonical H2A makes up about 50% of the H2A population in mononucleosomes purified with FLAG-H2A.Z or FLAG-macroH2A, suggesting that nucleosomes that contain these variants are asymmetric, containing one copy of canonical H2A (Figure 1C and Additional file 1: Table S1). Surprisingly, a small fraction of mononucleosomes were detected to possess both H2A.Z and macroH2A. On the other hand, analysis of H3.3-containing nucleosomes shows that canonical histone H2A is the most abundant form, followed by H2A.Z, and then finally macroH2A (Figure 1C and Additional file 1: Table S1).

We also quantified the relative ratios of H3.1 and H3.2 versus H3.3 in the FLAG-purified nucleosomes by using MS (Figure 1D). To do so, we utilized a peptide that was common in the H3.1 and H3.2 variants (a.a. 27 to 40) yet differed from the H3.3 variant by one amino acid (see the ‘Methods’ section). Therefore, we were not able to distinguish H3.1 from H3.2. From our calculations, H3.1 FLAG-purified nucleosomes contained histone H3 variants with a ratio of roughly 1:3 (H3.3:canonical H3) (Figure 1D and Additional file 1: Table S1). This suggests that most of the nucleosomes containing the variant H3.3 are asymmetric, containing one copy of H3.1 or H3.2 partnered with one copy of H3.3. This conclusion was further supported by the fact that approximately half of the FLAG H3.3-purified nucleosomes contained either H3.1 or H3.2 (Figure 1D and Additional file 1: Table S1). The most significant observation regarding the H3 variants was found in the FLAG macroH2A-purified nucleosomes; these nucleosomes contained only approximately 3% H3.3, suggesting that H3.3 is rarely found in nucleosomes containing the repressive macroH2A variant (Figure 1D and Additional file 1: Table S1). This result was further supported by the fact that Flag H3.3-purified nucleosomes contained very low levels of macroH2A (approximately 1%).

### Determination of histone PTM relative abundance

**Figure 2:**
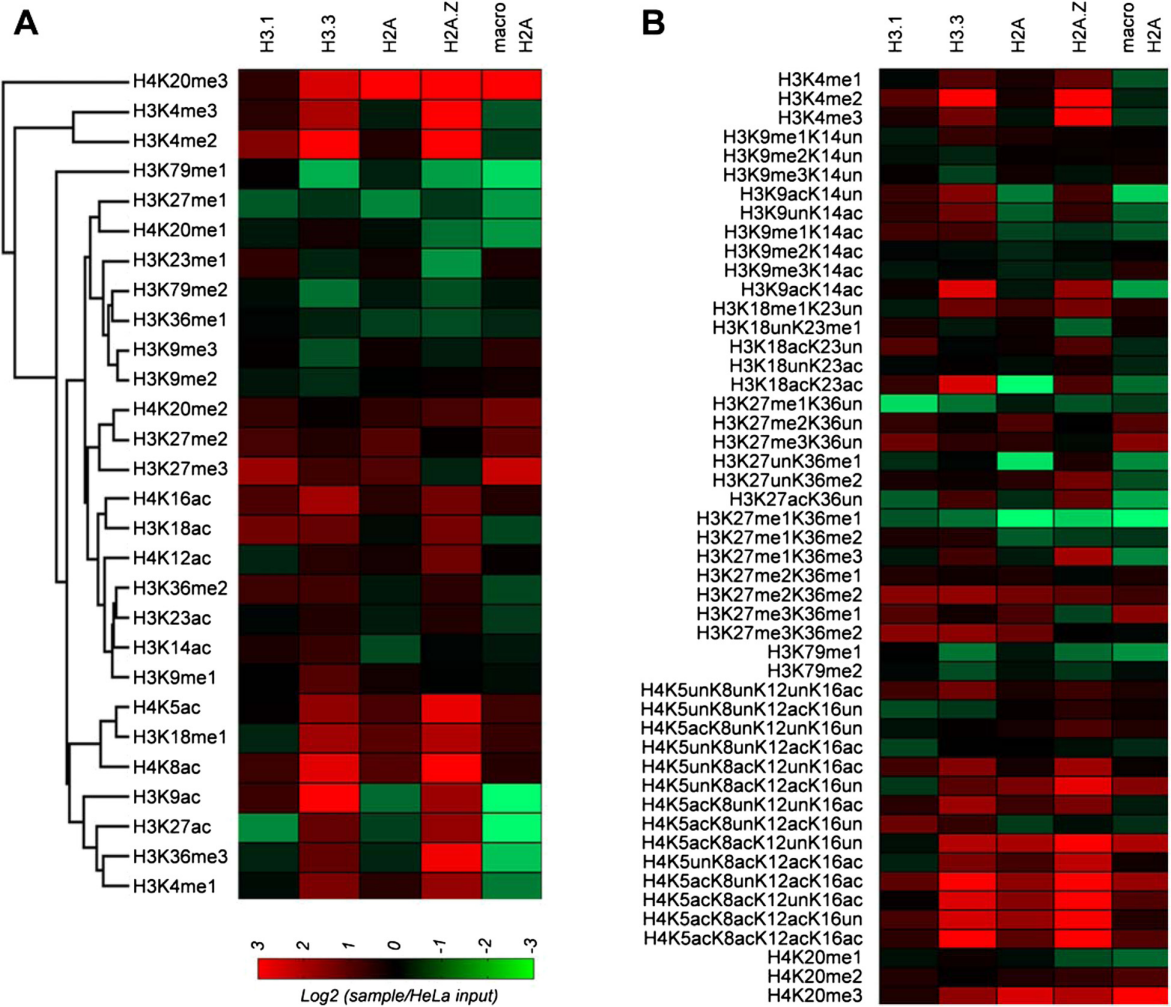
**Relative ratio of histone post-translational modifications in FLAG-IP experiments as compared to the global HeLa extract. (A)** Calculated relative abundance of single histone PTMs. Log_2_ ratio was calculated between each FLAG-IP sample (listed on top of the heat map) and the HeLa input. Single PTMs were sorted by common regulation into a hierarchical tree. **(B)** Log_2_ relative ratio of combinatorial histone PTMs, calculated using the same approach.

By using ChIP-MS results (Figure 2 and Additional file 2: Table S2), we investigated the relative abundance of histone PTMs in H2A.Z- and H3.3-containing mononucleosomes. Briefly, we observed an enrichment of active marks in such nucleosomes as compared to the global chromatin levels and, in particular, to nucleosomes containing the histone variant macroH2A. For instance, the activating mark H3K4me2 mark was highly enriched in nucleosome ChIPed with H3.3 or H2A.Z (11.5-fold in H3.3 and 19.8-fold in H2A.Z as compared with the genomic chromatin levels). This data was interesting considering that H2A.Z was not enriched in mononucleosomes purified with the H3 variant H3.3, indicating that H3.3 and H2A.Z may occupy distinct chromatin regions marked by H3K4me2. H3K4me3 was found to be enriched almost 30-fold in H2A.Z-purified nucleosomes as compared to global input (Figure 2A and Additional file 2: Table S2), which was consistent with previous observations that investigated active promoters [11]. A similar trend was observed for H3K36me3 in H3.3- and H2A.Z-purified nucleosomes, which was about 7% and 15% of the total histone H3, respectively. In genomic chromatin and macroH2A-containing nucleosomes, H3K36me3 was only 4% and 1%, respectively (Figure 2 and Additional file 2: Table S2). This was not surprising, as H3K36me3 is enriched downstream to the transcriptional start sites (TSSs) of active genes, which are the same genomic regions where H2A.Z is enriched [11]. Interestingly, H3.3-purified nucleosomes were less enriched in H3K36me3 than nucleosomes purified with H2A.Z, even though both H3.3 and H3K36me3 generally mark actively transcribed regions [11,18-21].

As compared to the total chromatin, both H3.3- and H2A.Z-purified nucleosomes were enriched for the activating marks H3K9ac and H3K27ac (4.3- and 2.4-fold changes for H3K9ac and 1.8- and 2.4-fold changes for H3K27ac, respectively) (Figure 2 and Additional file 2: Table S2). We also observed a dramatic enrichment of H4K16ac in nucleosomes purified with H3.3 (approximately 50%) and H2A.Z (approximately 40%) as compared to this modification in genomic chromatin (approximately 20%). This confirmed once again what we expected, as H3.3, H2A.Z, and H4K16ac are all enriched in gene bodies [28]. Moreover, H4K16ac is highly enriched in nucleosomes bound by the BET family bromodomain containing proteins (Brd2, Brd3, and Brd4), which are bound to and assist gene transcription by PolII [29]. The enrichment of H4K16ac was present in tandem with combinations of H4K5ac, H4K8ac, and H4K12ac in H3.3- and H2A.Z-purified nucleosomes (Figure 2B and Additional file 2: Table S2). Finally, the repressive mark H3K27me3 was enriched approximately threefold changes in macroH2A-purified nucleosomes as compared to genomic chromatin (approximately 18% in macroH2A-purified *vs.* approximately 6% in genomic or approximately 5% in H2A.1-purified nucleosomes). Moreover, we observed an inverse relationship between H3K4me2/3 and H3K27me3 on nucleosomes purified with either H2A.Z or macroH2A. Conversely, H3K27ac levels in macroH2A ChIPs were depleted (0.26%) as compared to the H3K27ac levels found in canonical H2A.Z ChIPs. Taken together, our data demonstrate that the trends of the major PTMs we observed in histones H3 and H4 were similar for H2A.Z and H3.3.

### Histone variant genome-wide profiles

**Figure 3:**
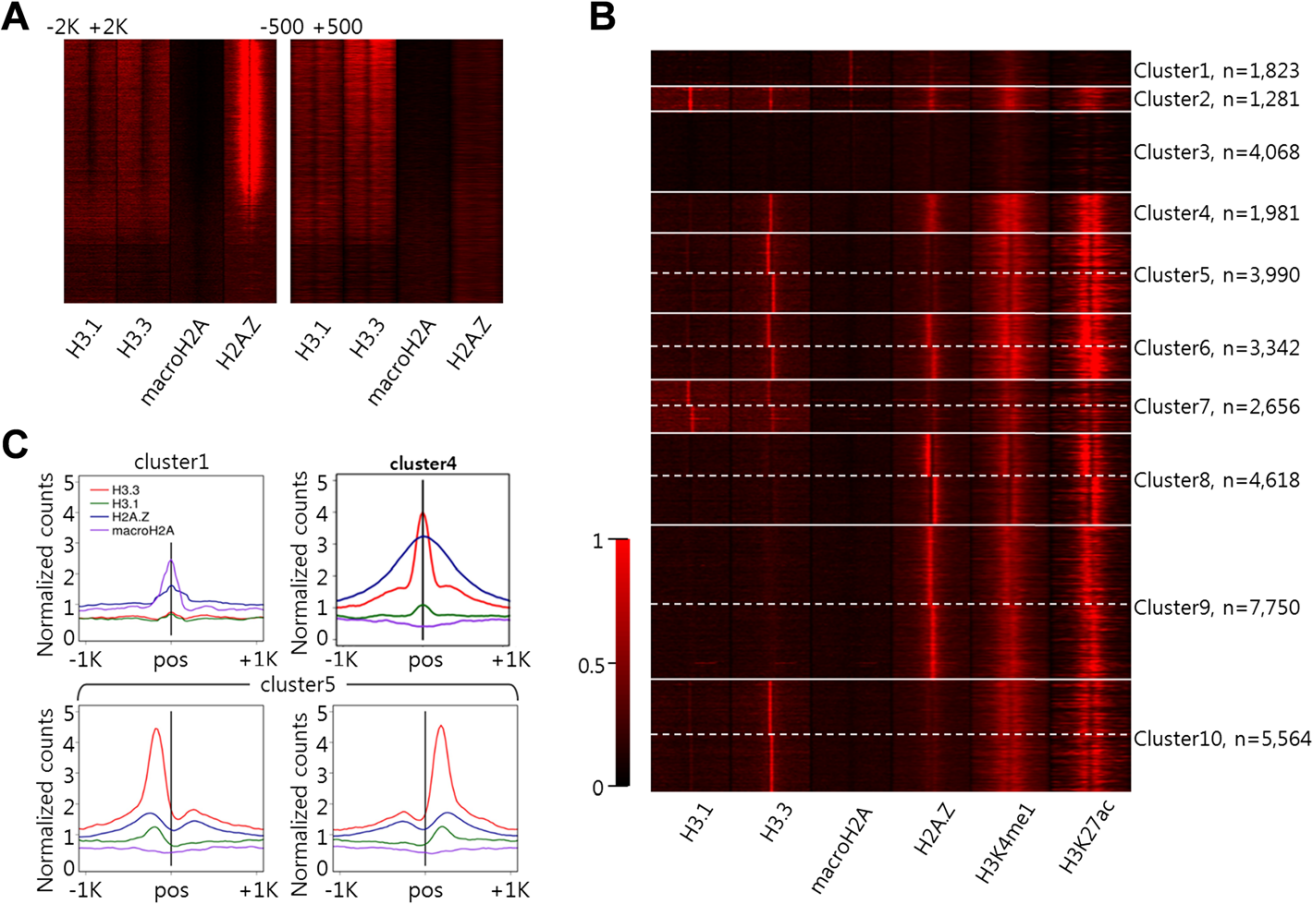
**Genomic profiles of histone variants. (A)** Distribution of ChIP-seq reads at annotated TSSs (±2 K) and TTSs (±500) and **(B)** at distal regulatory regions. We clustered DHSs located in the intergenic region. We identified 16 groups and rearranged them to 10 clusters based on their profiles. Various compositions of histone variants were found. Clusters 5 to 10 are composed of 2 mirroring groups. After clustering based on histone variants, we aligned histone modification. Histone variants are off-centered for the mirroring clusters (clusters 5 to 10), suggesting orientation at regulatory regions. **(C)** Symmetric and asymmetric profiles of histone variants. Clusters 1 and 4 show symmetric profiles with various compositions of histone variants. Cluster 5 shows mirroring asymmetric profile. All profiles for all clusters are shown in Additional file 3: Figure S2.

Our proteomic analyses further questioned how the co-occupying histone variants were represented in the genome. For this, we used deep sequencing approaches to map the FLAG-tagged histone variants in the genome. First, we asked how the histone variants are positioned around genes. After sorting the annotated Refseq genes based on their expression levels, we investigated the histone variant levels around the genes. H2A.Z was highly enriched around the annotated TSSs of active genes but absent in inactive genes (Figure 3A and Additional file 3: Figure S1). This was consistent with the previous genome-wide surveys [11,23]. The sharp enrichment of H2A.Z marked the two nucleosomes flanking the nucleosome-free regions (NFRs) at active promoters [11,30]. In the gene body, H2A.Z was absent regardless of their expression levels (Additional file 3: Figure S1).

Both H3.1 and H3.3 were depleted around the center of TSSs of active genes (Figure 3A and Additional file 3: Figure S1). These results differ from the previous study in mouse embryonic stem cells (mESCs) where H3.3 showed strong bimodal enrichment at the TSSs of active genes [16], but they are in agreement to the previous genome-wide study in HeLa cells [23]. At transcription termination sites (TTSs), we also found a depletion of H3.1 and H3.3 [31]. In general, H3.1 and H3.3 levels correlated with gene expression levels in the gene body. H3.3 levels were increased towards the 3′ end of the genes, consistent with previous studies [21,23]. While H2A.Z was sharply enriched around TSSs, the enrichments of H3.1 and H3.3 were modest. Also, we confirmed a negative correlation of the macroH2A with gene expression levels [32].

We then investigated if histone variants are enriched at promoter distal (>2 kbp from known TSSs) regulatory regions. For this, we retrieved the map of DNaseI hypersensitive sites (DHSs) in HeLa cells from ENCODE [33]. DHSs potentially demarcate regulatory elements, including promoters, enhancers, silencers, insulators, and locus control regions [34]. We identified a total of 94,600 DHSs using Homer [35]. Among them, 37,073 DHSs were located distal (>2 kbp) to the known TSSs, TTSs, and outside the body of the annotated mRNA and the long non-coding RNAs (lncRNAs). Monitoring the four histone variants (H2A.Z, macroH2A, H3.1, and H3.3) at the distal regulatory regions, we defined 16 groups (Figure 3B). We then further characterized such clusters by examining the histone PTMs H3K4me1 and H3K27ac, markers for enhancers [36,37]. Based on the proteomic study (Figure 2), we expected H2A.Z and H3.3 are with activating histone modification marks. Our results showed various combinations of histone variants at these distal regulatory regions. As expected, majority of the distal DHSs were enriched for H3.3 and/or H2A.Z as well as H3K27ac indicating that these histone variants are important for enhancer function. Also, we found clusters marked by H3.1 (clusters 2 and 7) or even with repressive macroH2A mark (cluster 1). Contrary to our proteomic results (Figure 2B), clusters 2 and 7 are enriched for H3.1 and activating H3K27ac mark at a certain level. The clustering results indicate diverse epigenetic codes composed of histone variants at distal regulatory regions.

### Histone variants have symmetric and asymmetric patterns at distal regulatory regions

Histone H3.3 and other histone variants were observed to have asymmetric profiles (Figure 3B, C and Additional file 3: Figure S2). The clusters 5 to 7 showed that H3.3 and H2A.Z were skewed to one side. We also checked the average profiles of p300, H3K27ac, and DHSs at each cluster. The H3K27ac profiles were enriched on the side where the H3.3 peak was located (Additional file 3: Figure S3) even though the skewness was less dramatic as compared to histone variant profiles. The DNaseI and histone acetyltransferase p300 profiles, marker for enhancers [37,38], were centered at the DHSs regardless of the pattern of the histone variants (Additional file 3: Figure S3), confirming that transcriptional co-factors are not biased to a single direction. Other transcription factors analyzed did not show asymmetric patterns either (data not shown), further indicating that the skewness in the histone variant profiles was independent from transcription factors and their co-factors.

**Figure 4:**
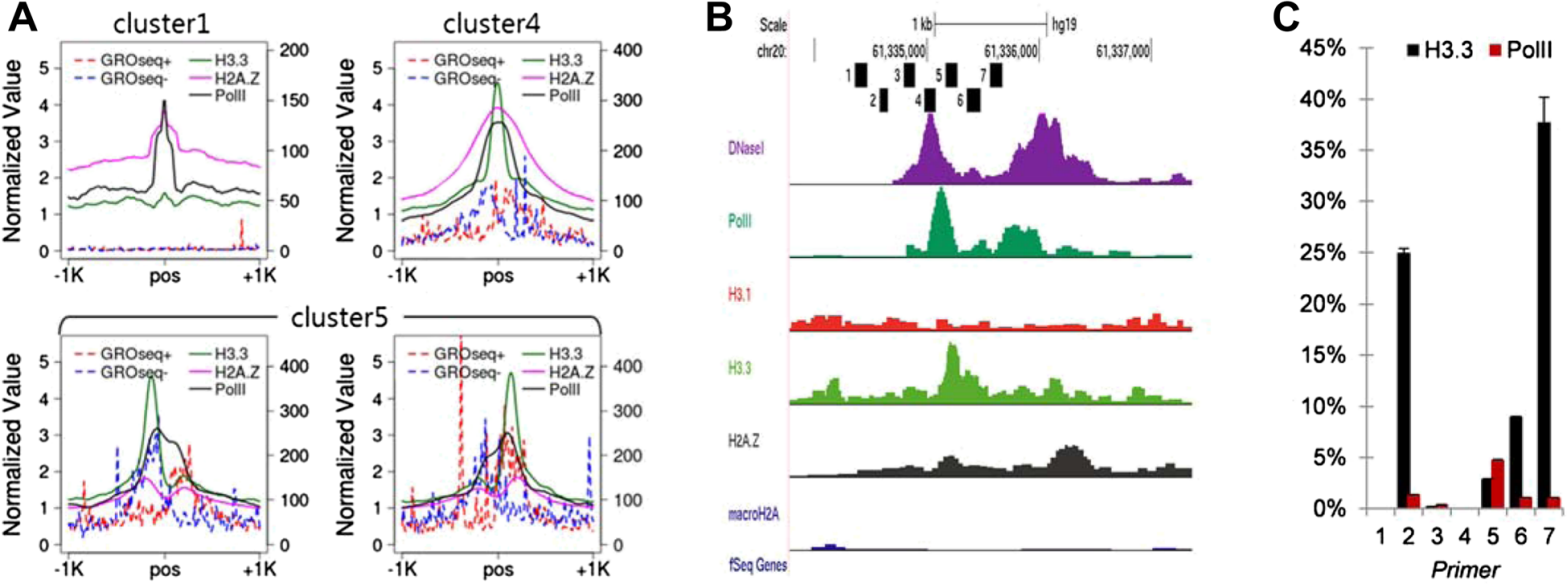
**Histone variants are associated with PolII orientation. (A)** The profiles of H3.3 and H2A.Z are associated with the pattern of PolII and enhancer transcripts. For symmetric clusters, PolII is located at the center. For asymmetric clusters, PolII is skewed to the direction of the peaks of histone variants. Transcripts at enhancers show bidirectional patterns. Strand-specific transcripts are stronger in the asymmetric clusters. **(B)** The screenshot of histone variant around DNaseI. H3.3 is enriched more to the right side of the peak of DNaseI. PolII has its peak to the right side of DNaseI. **(C)** ChIP-qPCR validation on the seven regions. PolII enrichment is skewed towards the right side.

To further investigate the association of histone variants with gene regulation, we examined PolII occupancy, which was found to be skewed towards the peak of H3.3 and H2A.Z (clusters 5 to 10) (Figure 4A and Additional file 3: Figure S4). In clusters 1 to 4, PolII peaked at the center of DHSs. In these clusters, H3.3 either peaked directly over the DHSs (clusters 2 and 4) or showed no enrichment over the entire 2Kb region surrounding the DHSs (clusters 1 and 3). In clusters 5, 6, 7, and 10, PolII was skewed towards the peak of H3.3. This suggests that the position of H3.3 is related to transcriptional orientation at distal regulatory regions. Figure 4B shows an example of skewness of H3.3 in association with PolII orientation. H3.3 is located on a side of the DHS, and PolII peak was observed between the DHS and H3.3 peak, as shown in the profile. We validated this observation by performing ChIP-qPCR of the five regions around the DHSs. The qPCR experiment confirmed that PolII and H3.3 were not symmetric at a potential enhancer and skewed towards the same direction (Figure 4C).

### Role of chromatin domains containing studied histone variants

We investigated the enhancer activities of these potential regulatory using the global run-on sequencing (GRO-seq) data in HeLa cells [39]. GRO-seq can measure the transcription levels at enhancers (or eRNA [40,41]). All clusters showed bidirectional transcripts in their eRNA profiles. Especially, we observed modest bias of the strand-specific transcripts that matched with the PolII orientation in the asymmetric clusters (Figure 4A and Additional file 3: Figures S4 and S5). The enhancer activity and the PolII levels of the cluster with macroH2A (cluster 1) were very weak. Actually, the eRNA levels of cluster 1 were even smaller than cluster 3 (*P* value = 3.4e − 37), where we did not find any strong signal of histone variants. This further confirmed the repressive role of macroH2A at distal regulatory regions. Both clusters 5 and 10 were enriched for H3.3, but eRNA levels were significantly larger in cluster 5 where the skewness was more pronounced (*P* value = 1.3e − 13). We also observed stronger eRNA levels for cluster 8 than cluster 9, where skewness was more strongly observed (*P* value = 6.4e − 11). Collectively, these suggest that skewed histone variants facilitate enhancer activity.

DHSs represent open chromatin regions in the genome. Unexpectedly, clusters 2 and 4 had histone variants enriched at DHSs, which are potential nucleosome-free regions. Interestingly, the dip in the bimodal pattern for H3K27ac was rather shallow or lost for clusters 2 and 4 (Additional file 3: Figure S3). Previous studies have reported that nucleosome core particles (NCPs) containing both H3.3 and H2A.Z are unstable and that such NCPs were enriched at nucleosome-depleted regions [23,42]. Thus, clusters 2 and 4 may represent an example of the unstable histone variants at DHSs.

Finally, we investigated the occupancy of transcription factors into the clusters we identified. We calculated the hyper-geometric *P* value using the peak information for each cluster. Interestingly, we observed that transcription factors prefer specific epigenetic environments (Additional file 3: Figure S6); clusters 1, 2, 3, and 7 were uniquely populated with specific factors such as CTCF and Zzz3. CTCF is involved in many functions including transcriptional activation/repression, insulation, imprinting, and forming higher-order structures [43]. CTCF binding was significantly observed at cluster 3, where the levels of the activating histone marks H3K4me1 and H3K27ac were depleted (Additional file 3: Figure S5), presumably associated with the insulator function. Interestingly, cluster 7 was uniquely enriched for CTCF, suggesting that the enrichment of H3.1 is designated to a specific function of CTCF. We also found Zzz3 significantly enriched in clusters 1 and 2 as compared to the other clusters, which are potentially containing unstable nucleosome at DHSs (Additional file 3: Figure S6). Overall, this suggests that Zzz3 may associate with other factors when forming closed chromatin structure.

## Conclusions

Histone non-canonical variants play a major regulatory role in mammalian genomes. For example, H2A.Z is enriched at active promoters as well as active enhancers marked by H3K27ac, and H3.3 was found enriched at the peaks of H2A.Z [16]. In this paper, we dissected the composition of histone variants using both proteomics and genomics strategies. Our quantitative MS results revealed that activating histone PTMs were highly enriched on H2A.Z-containing and H3.3-containing mononucleosomes, while such marks were generally depleted in macroH2A-containing mononucleosomes. Conversely, macroH2A-containing mononucleosomes were enriched for repressive histone PTMs.

We further investigated the distribution of histone variants in the genome especially at distal regulatory regions. We identified diverse compositions of four histone variants at potential regulatory regions. Besides H3.3 and H2A.Z, which have been known to be enriched at regulatory regions [16,21], we additionally found regulatory regions enriched in H3.1 or macroH2A. The regulatory regions enriched for macroH2A were depleted for active histone marks and enhancer activities, suggesting a repressive role for this element (cluster 1, Figure 3C). H3.1 was observed in two clusters: one in the closed chromatin structure (cluster 2) and one with strong enrichment of CTCF. This evidenced that histone H3.1 has still several unknown functions at distal regulatory regions. Unexpectedly, we also found histone variants at the center of DHSs, potential nucleosome-free regions. Specifically, we observed the centers of some DHSs enriched for H2A.Z and H3.3 (clusters 2 and 4). These regulatory elements may be associated with unstable nucleosomes containing H3.3/H2A.Z double variant at DHS [23].

Importantly, we found asymmetric patterns of histone variants. In general, symmetric bimodal patterns have been accepted as profile for activating histone marks [37,44,45]. Our results showed a large portion of regulatory regions with asymmetric patterns. These regulatory regions were associated with the skewed patterns of the activating mark H3K27ac. Besides, the asymmetric patterns of histone variants were associated with PolII occupancy. Taken together, this demonstrated that skewed histone variants were not just noise, but such deposition dictates the direction of PolII movement.

Enhancers were originally defined as remote elements that increase transcription independently of their orientation [46,47]. However, some groups already identified a number of enhancer groups with asymmetric histone modification patterns [48]. Other computational models found asymmetric H2A.Z and nucleosome occupancies at CTCF binding sites [49,50]. More directly, nascent RNAs at enhancers show both bidirectional and unidirectional transcripts [51]. The 5′ ends of capped RNAs detected by Cap Analysis of Gene Expression (CAGE) in HeLa cells confirmed unidirectional transcripts at enhancers [39]. Nascent RNAs are closely associated with RNA polymerase [52,53]. The asymmetric patterns of eRNA as well as the skewed PolII occupancy in our study suggest that enhancers have directional information. For example, experiments that flipped enhancer sequences changed the activity of promoter in the luciferase assay [54], showing that directional information of the flanking regions around distal regulatory regions is important for gene regulation.

PolII orientation is clearly defined at promoters, where transcriptional orientation defines the asymmetric epigenetic pattern of H3K4me3 towards the direction of transcription [11]. A remarkable observation was made for bidirectional as well as unidirectional promoters in association with histone variants. H2A.Z at active promoters show strong upstream as well as downstream peaks in human and yeast [11,17,30,55], but not in flies [56] or *Arabidopsis* [57]. The presence of upstream H2A.Z nucleosomes seen in some organisms correlates with bidirectional transcription in yeast and mammals [53,58]. This suggests that histone variants are associated with transcriptional direction. At promoters, moreover, the nucleosome located to the transcriptional direction blocks the movement of PolII [59]. Depletion of H2A.Z from a nucleosome position resulted in a higher barrier to PolII [59]. Enhancers bare similar characteristics with promoters. Besides eRNAs, some enhancers are even with TATA box sites [39]. The nucleosome at distal regulatory region may block the movement of PolII as at promoters. H3.3 as well as H2A.Z may work to remove the barrier for PolII at distal regulatory regions. We additionally investigated the histone variants as well as PolII profiles at active promoters (Additional file 3: Figure S7). The genome-wide profile showed that the PolII and H3.3 profiles at enhancers are strikingly similar to the profile of H2A.Z and PolII at promoters.

**Figure 5:**
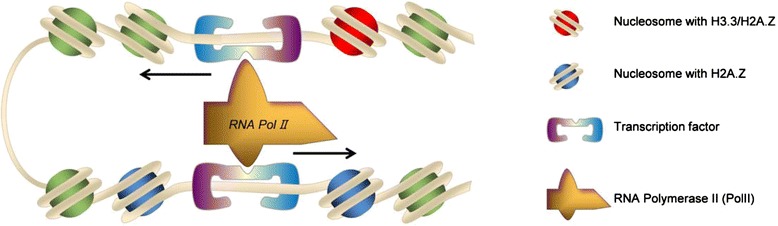
**A possible model for gene regulation associated with H3.3.** The nucleosome at enhancer located to the transcription direction is with histone variants to facilitate the movement of PolII.

In conclusion, why do enhancers have orientation? A DNA looping model has been suggested where promoter-enhancer interactions facilitate gene transcription [60-62]. These observations questioned the transcriptional mechanism associated with histone variants and PolII. By forming a looping structure, PolII needs to have a preference for its movement as it needs to move towards the transcription orientation of the associated gene (Figure 5). The nucleosome on one side of the regulatory region may block the movement of PolII at distal regulatory regions. H3.3 and H2A.Z may help the movement of PolII by destabilizing the nucleosome that blocks the movement of PolII.

## Methods

### Chromatin immunoprecipitation of histone variants

HeLa S3 cells stably expressing either FLAG-tagged canonical histone H3, H3.3, canonical histone H2A, and H2A.Z were grown in suspension in Joklik media containing 10% newborn calf serum (Hyclone, GE Healthcare, Little Chalfont, UK), 1% GlutaMAX (Invitrogen, Carlsbad, CA, USA), and 1% penicillin-streptomycin, and cells were harvested at log phase. Nuclei were isolated, mononucleosomes were subsequently obtained from these cell lines, and ChIP experiments were performed as described [29]. Briefly, cells were lysed in hypotonic TMSD buffer to isolate nuclei, which were then digested with micrococcal nuclease. The resulting mononucleosomes (Figure 1A, B) were immunoprecipitated with anti-FLAG M2-agarose beads (Sigma-Aldrich, St. Louis, MO, USA) and eluted with FLAG peptide (Sigma-Aldrich, St. Louis, MO, USA).

### Preparation of histones for mass spectrometry

Histone samples, from both ChIP input and ChIP elutions, were prepared as described previously [27], with the exception that the acid extraction step was simply replaced by a boiling step for the ChIP elutions. Briefly, digestion was performed as follows: derivatization reagent was prepared by mixing propionic anhydride with 2-propanol in the ratio 1:3 (*v*/*v*) and added to the histone sample in the ratio of 1:2 (*v*/*v*) for 15 min at 37°C. This reaction was performed twice to obtain complete labeling. Histones were then digested with trypsin (enzyme:sample ratio of 1:20, overnight at room temperature) in 50 mM NH_4_HCO_3_. After digestion, the derivatization reaction was performed again twice to derivatize peptide N-termini. Samples were desalted by using C18 Stage-tips, and resuspended in 0.1% formic acid for LC-MS analysis.

### Quantitative mass spectrometry

Histone samples were analyzed using nano liquid chromatography-tandem mass spectrometry (nanoLC-MS/MS) essentially as described previously [27]. Briefly, nanoLC was configured with a 75 μm ID × 17 cm Reprosil-Pur C18-AQ (3 μm; Dr. Maisch GmbH, Germany) nano-column using an EASY-nLC nanoHPLC (Thermo Scientific, Odense, Denmark). Detection was performed by using an Orbitrap XL mass spectrometer (Thermo Scientific, Odense, Denmark). Peak area was extracted from raw files by using our in-house software EpiQuant. The relative abundance of a given PTM was calculated by dividing its intensity by the sum of all modified and unmodified peptides sharing the same sequence. All raw files are available at the Chorus database (https://chorusproject.org).

### Determination of variant histone relative abundance

In order to determine the fraction of canonical histone H2A, macroH2A, and H2A.Z histones that comprises fH3.1-, fH3.3-, fH2Ac-, and fH2A.Z-containing mononucleosomes, we first quantified four peptides within the canonical histone H2A protein, namely the abundances of 4-11 (GKQGGKAR; H2Ac_1_), 12-17 (AKAKTR; H2Ac_2_), 21-29 (AGLQFPVGR; H2Ac_3_), and 82-88 (HLQLAIR; H2Ac_4_) peptides in each sample, which we refer to as reference peptides. Next, we quantified the abundances of the variant-specific peptides, namely the 36-42 canonical histone H2A peptide (KGNYAER; H2Ac), the 4-14 macroH2A peptide (GGKKKSTKTSR), and the 1-19 H2A.Z peptide (AGGKAGKDSGKAKTKAVSR). We then normalized a given variant-specific peptide such as the 1-19 H2A.Z peptide over each of the four references to attain four separate ratios, namely a common H2A.Z numerator over four different denominators (that is, H2A.Z/H2Ac_1_, H2A.Z/H2Ac_2_, H2A.Z/H2Ac_3_, H2A.Z/H2Ac_4_). We repeated this normalization for the other variant-specific peptides. To determine the relative ratios of the H3 variants, we quantified a peptide common to H3.1 and H3.2 27-40 (KSAPATGGVKKPHR) and compared it directly to the H3.3 peptide 27-40 (KSAPSTGGVKKPHR) that differs by only a single amino acid at residue 31 (alanine in H3.1 and H3.2 and serine in H3.3).

### Genomic data processing

All ChIP-seq tags were aligned to the human genome hg19 using Bowtie [63] with options ‘-v 2 -m 1 --best --strata,’ and all of the redundant tags were removed before downstream analysis. All ChIP-seq data were normalized to 10 reads per kilobase per million mapped reads (RPKM) [64]. Besides H3.1 and H3.3, we used ChIP-seq data for macroH2A [32], H2A.Z H3K4me1, and H3K27ac [33]. Additional file 1: Table S1 summarizes all the data we integrated in our study. Besides, we used the ChIPseq data for various transcription factors (TFs) and co-factors from ENCODE database [33] to investigate enriched binding for each cluster. We used the processed peak files deposited in the ENCODE database for this analysis. Hyper-geometric *P* values were calculated to identify enriched occupancy for TFs and co-factors. We used Refseq annotated genes (hg19) and sort them based on expression level from RNAseq data in HeLa cells [33]. DHSs were identified using Homer [35]. We excluded DHSs at promoter or body of annotated genes in GENCODE v19 [65]. Clustering was performed using the obtained distal DHSs. For clustering analysis, we used MeV V4.8 [66] and applied the *K*-means clustering algorithm to the ChIP-seq data of histone variants (H3.1, H3.3, macroH2A, and H2A.Z) using the Pearson correlation with absolute distance as a metric (*K* = 10). Clusters were rearranged to group the mirroring images. Average profiles were obtained using the Homer package [35].

### Data availability

The ChIP-seq data from this study are available at the NCBI Gene Expression Omnibus (GEO; http://www.ncbi.nlm.nih.gov/geo/) under accession number (GSE64652).

## Additional files

Additional file 1: Table S1. The ChIP-seq data sets included in the study. Previous ChIP-Seq data sets that were used and compared with our results. Additional file 2: Table S2. Relative abundance of histone modifications from FLAG-IP-purified samples. Quantification normalized histone PTM relative abundances from the mass spectrometry data. Additional file 3:
**Figure S1.** The averaged profiles of histone variants in association with gene expression. **Figure S2.** Histone variants’ codes and their shapes. **Figure S3.** Histone variants and other factors. **Figure S4.** PolII is enriched to the direction of H3.3. **Figure S5.** Comparison of eNA levels and PolII levels. **Figure S6.** Transcription factors and co-factors enriched at each cluster significantly enriched at each cluster. **Figure S7.** The averaged profiles of H3.3, H2A.Z, and PolII at the TSSs of active genes.

## Abbreviations

CAGE: cap analysis of gene expression
ChIP: chromatin immunoprecipitation
ChIP-MS: ChIP followed by mass spectrometry
ChIP-seq: ChIP followed by next-generation sequencing
DHS: DNaseI hypersensitive site
eRNA: enhancer RNA
GEO: gene expression omnibus
GRO-seq: global run-on sequencing
lncRNA: long non-coding RNA
mESC: mouse embryonic stem cell
NCP: nucleosome core particle
NFR: nucleosome free regions
PTM: post-translational modification
PolII: RNA polymerase II
RPKM: reads per kilobase per million mapped reads
TF: transcription factor
TSS: transcriptional start site
TTS: transcription termination site
