# Supplementary material for: Proteogenomics analysis reveals specific genomic orientations of distal regulatory regions composed by non-canonical histone variants
Source: Epigenetics Chromatin. 2015 Apr 10;8:13. doi: 10.1186/s13072-015-0005-9 (PMC4397702; doi:10.1186/s13072-015-0005-9)
Supplement: Additional file 1: Table S1. — The ChIP-seq data sets included in the study. Previous ChIP-Seq data sets that were used and compared with our results. [file 13072_2015_5_MOESM1_ESM.docx]

Table S1. **The ChIP-seq data set included in the study**

| Antibody | Data type | reference | GEO ID |
| --- | --- | --- | --- |
| Flagged H3.1 and H3.3 | ChIP-seq |  | GSE64652 |
| Flagged macroH2A | ChIP-seq | ([Tolstorukov et al. 2012](#_ENREF_3)) | GSM937967 |
| H2A.Z | ChIP-seq | ([The ENCODE Project Consortium 2012](#_ENREF_2)) | GSM1003483 |
| H3K4me1 | ChIP-seq | ([The ENCODE Project Consortium 2012](#_ENREF_2)) | GSM798322 |
| H3K27ac | ChIP-seq | ([The ENCODE Project Consortium 2012](#_ENREF_2)) | GSM733684 |
| PolII | ChIP-seq | ([The ENCODE Project Consortium 2012](#_ENREF_2)) | GSM733759 |
| DNaseI | DNase-seq | ([The ENCODE Project Consortium 2012](#_ENREF_2)) | GSM736510 |
| RNA-seq | RNA-seq | ([The ENCODE Project Consortium 2012](#_ENREF_2)) | GSM591682 |
| GROseq | GROseq | ([Andersson et al. 2014](#_ENREF_1)) | GSE62046 |

Andersson R, Refsing Andersen P, Valen E, Core LJ, Bornholdt J, Boyd M, Heick Jensen T, Sandelin A. 2014. Nuclear stability and transcriptional directionality separate functionally distinct RNA species. *Nature communications* **5**: 5336.

The ENCODE Project Consortium. 2012. An integrated encyclopedia of DNA elements in the human genome. *Nature* **489**(7414): 57-74.

Tolstorukov MY, Goldman JA, Gilbert C, Ogryzko V, Kingston RE, Park PJ. 2012. Histone variant H2A.Bbd is associated with active transcription and mRNA processing in human cells. *Mol Cell* **47**(4): 596-607.
