# Supplementary material for: Proteogenomics analysis reveals specific genomic orientations of distal regulatory regions composed by non-canonical histone variants
Source: Epigenetics Chromatin. 2015 Apr 10;8:13. doi: 10.1186/s13072-015-0005-9 (PMC4397702; doi:10.1186/s13072-015-0005-9)

Proteogenomics analysis reveals specific genomic orientations of distal regulatory regions composed by non-canonical histone variants – Supplementary material

Kyoung-Jae Won[1,2,6,*], Inchan Choi[1,2,5,*], Gary LeRoy[3,*], Barry M. Zee[3,4], Simone Sidoli[4], Michelle Gonzales-Cope[3,4], and Benjamin A. Garcia[4,6]

[1]The Institute for Diabetes, Obesity, and Metabolism,

[2]Department of Genetics, Perelman School of Medicine, University of Pennsylvania, Philadelphia, Pennsylvania 19104, USA

[3]Department of Molecular Biology, Princeton University, Princeton, NJ 08544, USA

[4]Epigenetics Program, Department of Biochemistry and Biophysics, Perelman School of Medicine, University of Pennsylvania, Philadelphia, PA, 19104, USA

[5]Dept. of Agricultural Biotechnology, National Academy of Agricultural Science, 370 Nongsaengmyeong-ro, Wansan-gu, Jeonju-si, Jeollabuk-do, 560-500 South Korea

[*]These authors contributed equally.

[6]To whom correspondence should be addressed

Email: wonk@mail.med.upenn.edu, phone: 1-215-866-8366

bgarci@mail.med.upenn.edu, Phone: 1-215-573-9423, Fax: 215-898-4217

Figure S1: **The averaged profiles of histone variants in association with gene expression.** We plotted the levels of histone variants of all, highly active (top 3000) and inactive (bottom 5000) genes in their promoter (-2k~1k of TSSs), gene body and the 3'end (-1kb~1kbp of TTSs).

Figure S2: **Histone variants codes and their shapes.** Histone variants showed various combination as well as symmetric and asymmetric shape. Clusters 1-4 have symmetric pattern, while clusters 5-10 have asymmetric pairs.

Figure S3: **Histone variants and other factors**. We investigated p300, H3K27ac and DNaseI hypersensitivity. P300 showed symmetric pattern regardless of H3.3 but histone acetylation was enriched more with H3.3 peak.

Figure S4: **PolII is enriched to the direction of H3.3.** For symmetric clusters PolII was located at the center. For asymmetric clusters, PolII is skewed to the direction of the peak of histone variants. Transcripts at enhancers showed bidirectional patterns. Strand-specific transcripts are stronger in the assymmetric clusters.

Figure S5: **Comparison of eNA levels and PolII levels.** The eRNA levels show the enhancer activity. Symmetric enhancers have similar eRNA levels for the plus and the minus strand. eRNA levels were stronger in the strand supported by the transcription orientation.

Figure S6: **Transcription factors and co-factors enriched at each cluster significantly enriched at each cluster.** We investigated the binding of transcription factors and co-factors. –log10 p-value is shown using hypergeometric distribution.

Figure S7: **The averaged profiles of H3.3, H2A.Z and PolII at the TSSs of active genes.** The levels of H3.3, H2A.Z and PolII of highly active (top 3000) genes in their promoter (-2k~2k of TSSs).

Table S1: **The ChIP-seq data sets included in the study.**

Table S2: **Relative abundance of histone modifications from FLAG-IP purified samples.** The table includes the calculated relative abundance of combinatorial and single histone PTMs from HeLa cells (input) and the purified nucleosomes containing the FLAG tagged histone variants.

Figure S1

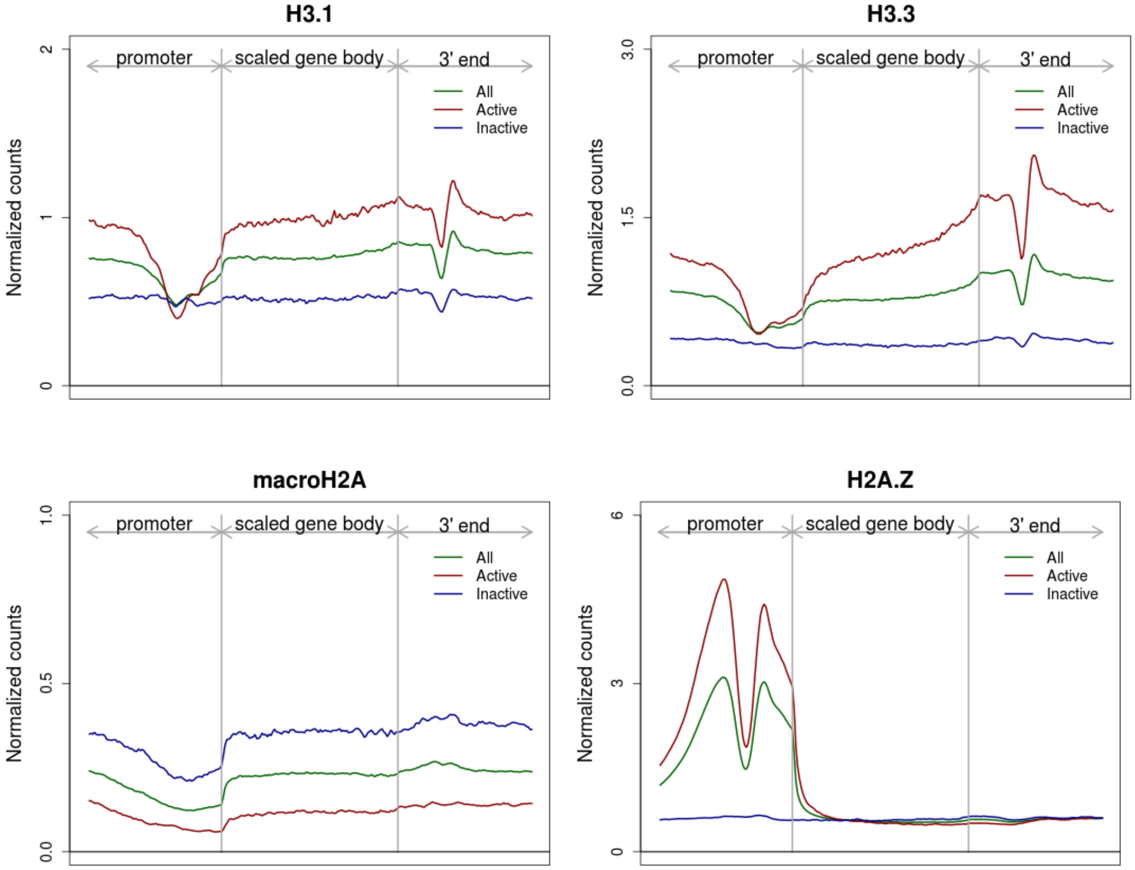
H3.1
promoter
scaled gene body
3' end
All
Active
Inactive
Normalized counts
0
1
2
H3.3
promoter
scaled gene body
3' end
All
Active
Inactive
Normalized counts
0.0
1.5
3.0
macroH2A
promoter
scaled gene body
3' end
All
Active
Inactive
Normalized counts
0.0
0.5
1.0
H2A.Z
promoter
scaled gene body
3' end
All
Active
Inactive
Normalized counts
0
3
6

Figure S2

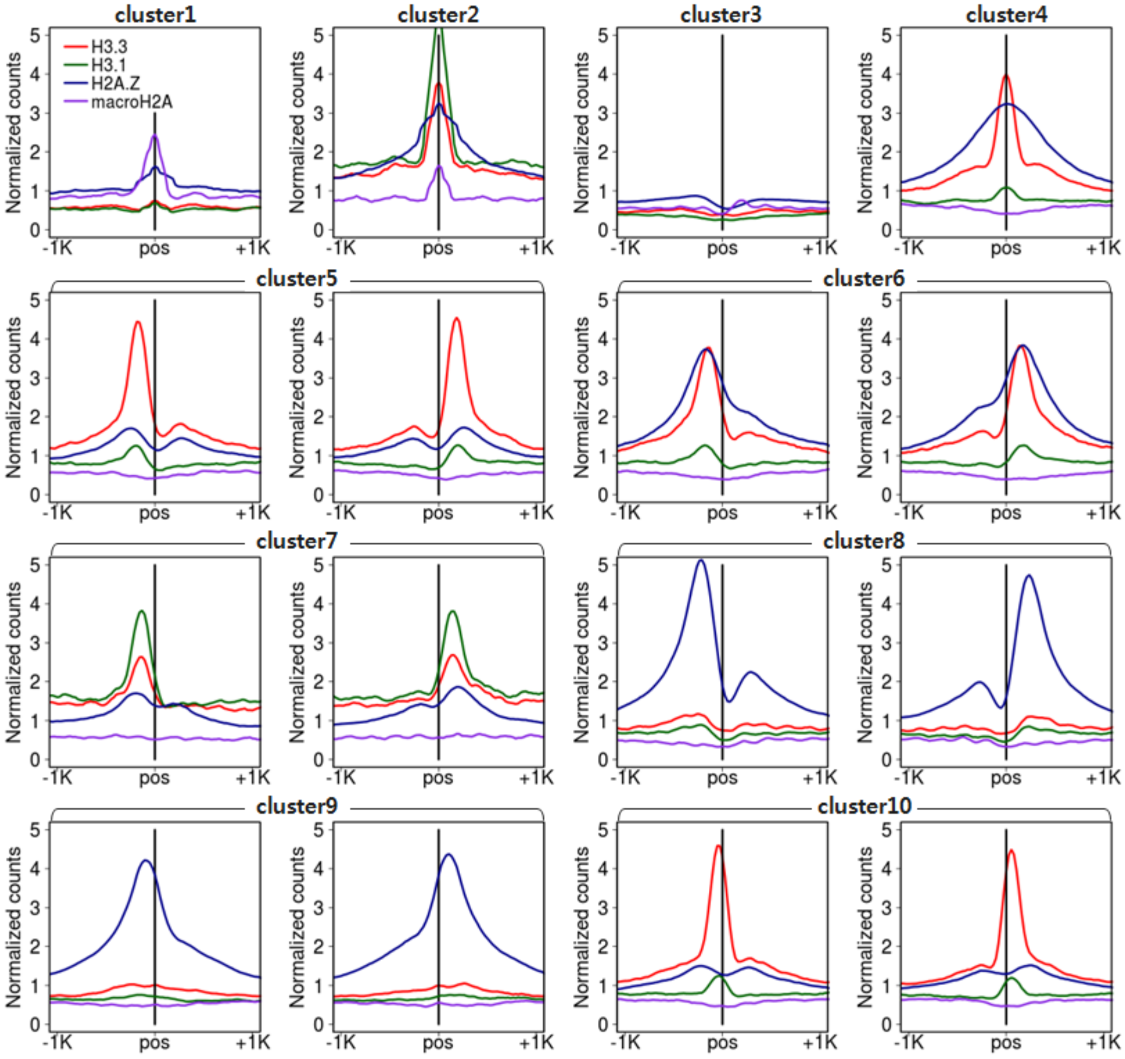
cluster1
cluster2
cluster3
cluster4
cluster5
cluster6
cluster7
cluster8
cluster9
cluster10
H3.3
H3.1
H2A.Z
macroH2A
Normalized counts
-1K
pos
+1K

Figure S3

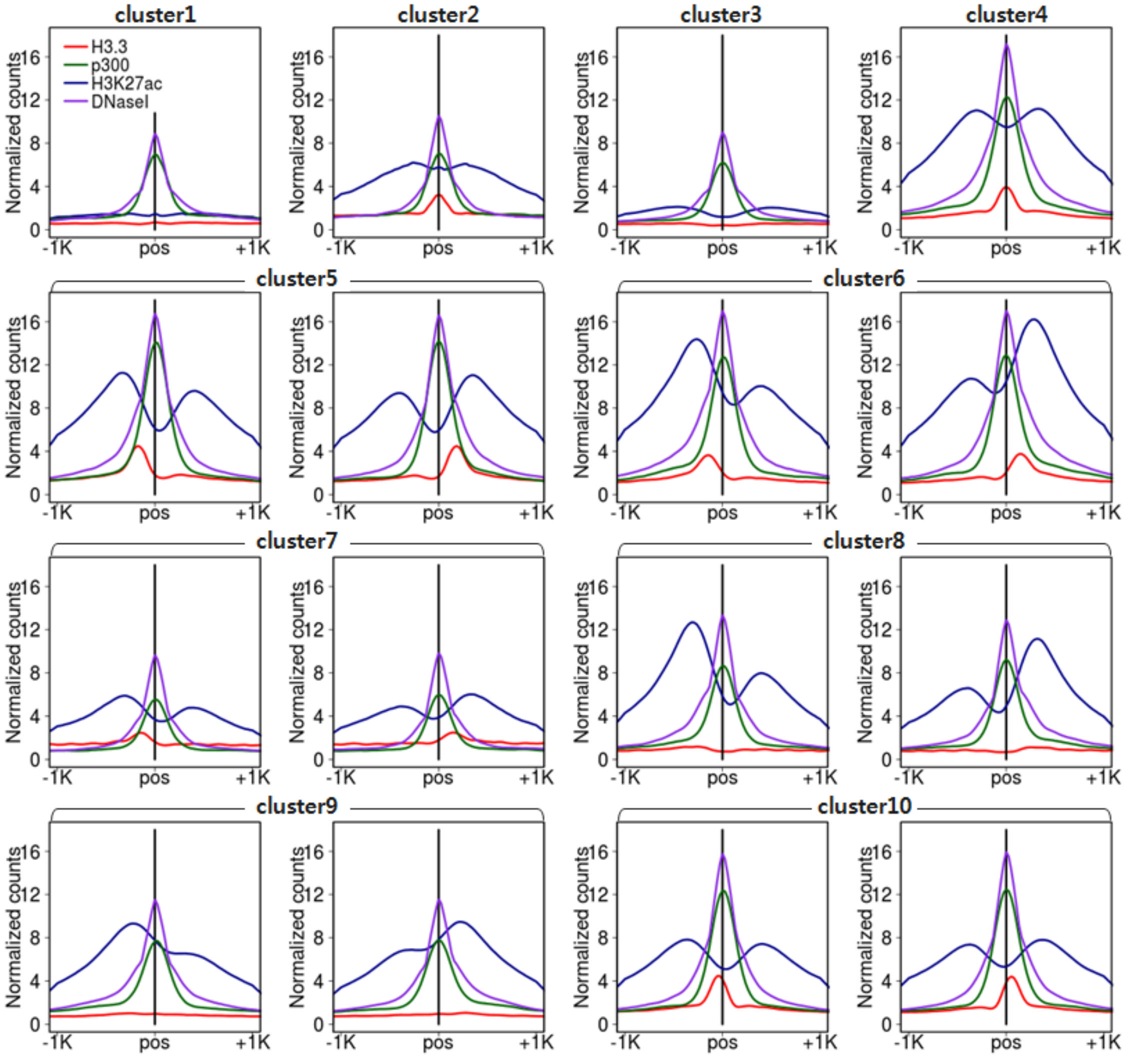

cluster1
cluster2
cluster3
cluster4
cluster5
cluster6
cluster7
cluster8
cluster9
cluster10
H3.3
p300
H3K27ac
DNaseI
Normalized counts
0
4
8
12
16
-1K
pos
+1K

Figure S4

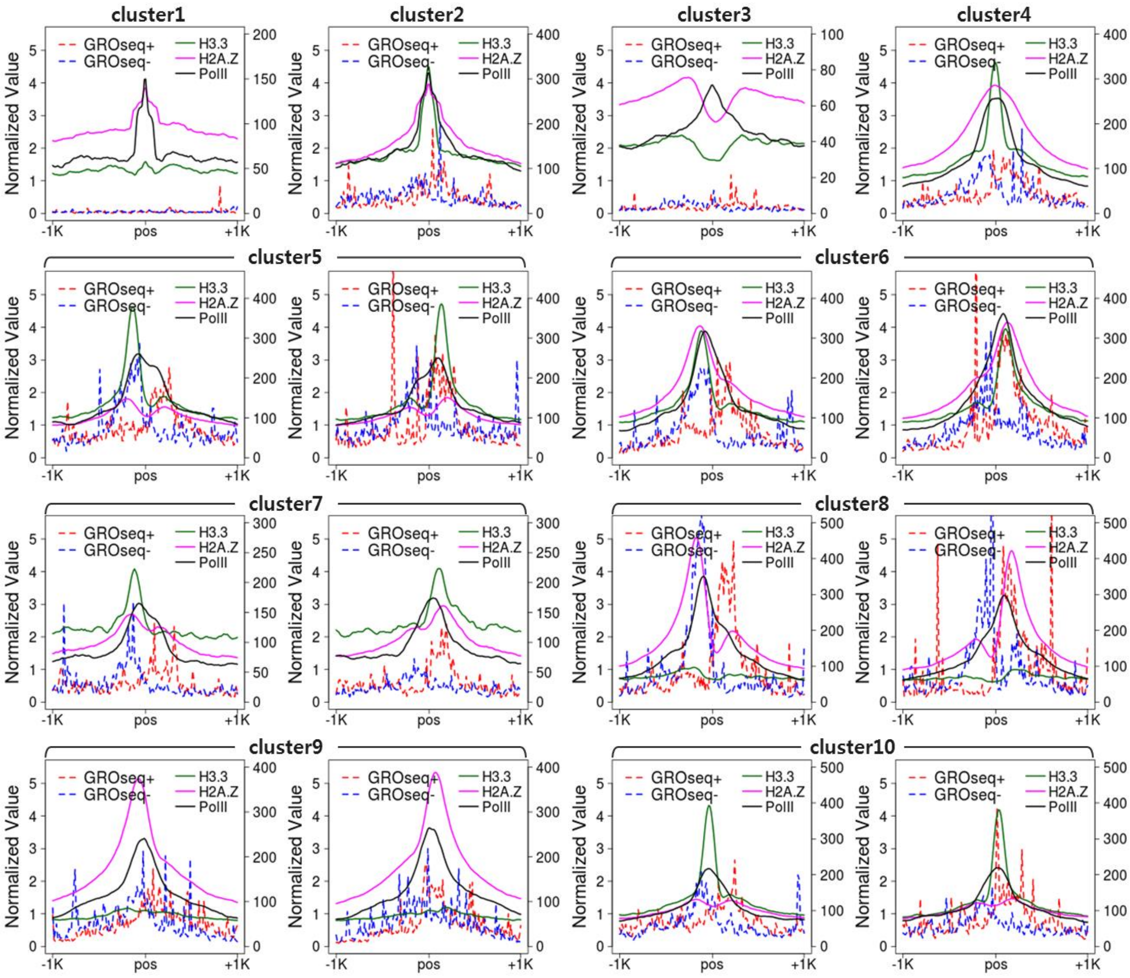

cluster1
cluster2
cluster3
cluster4
cluster5
cluster6
cluster7
cluster8
cluster9
cluster10
GROseq+
GROseq-
H3.3
H2A.Z
PolII
Normalized Value
pos
-1K
+1K

Figure S5

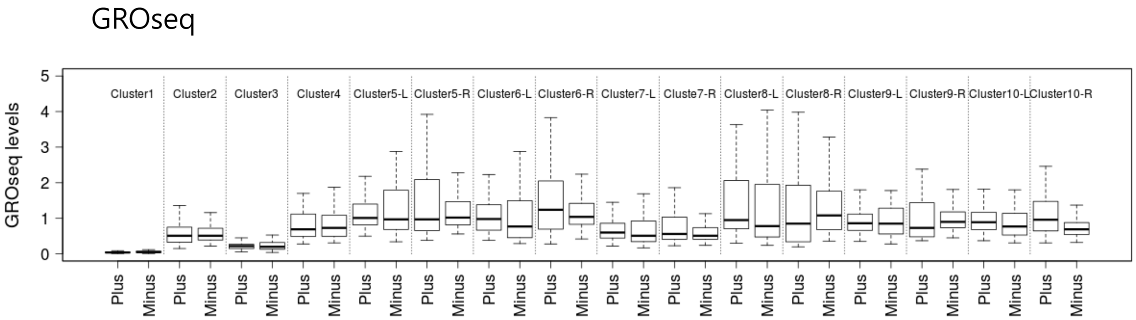
GROseq
GROseq levels
0
1
2
3
4
5
Cluster1
Cluster2
Cluster3
Cluster4
Cluster5-L
Cluster5-R
Cluster6-L
Cluster6-R
Cluster7-L
Cluste7-R
Cluster8-L
Cluster8-R
Cluster9-L
Cluster9-R
Cluster10-L
Cluster10-R
Plus
Minus

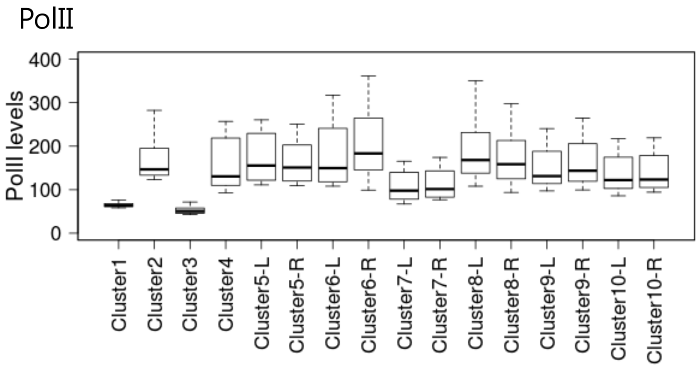
PolII
PolII levels
0
100
200
300
400
Cluster1
Cluster2
Cluster3
Cluster4
Cluster5-L
Cluster5-R
Cluster6-L
Cluster6-R
Cluster7-L
Cluster7-R
Cluster8-L
Cluster8-R
Cluster9-L
Cluster9-R
Cluster10-L
Cluster10-R

Figure S6

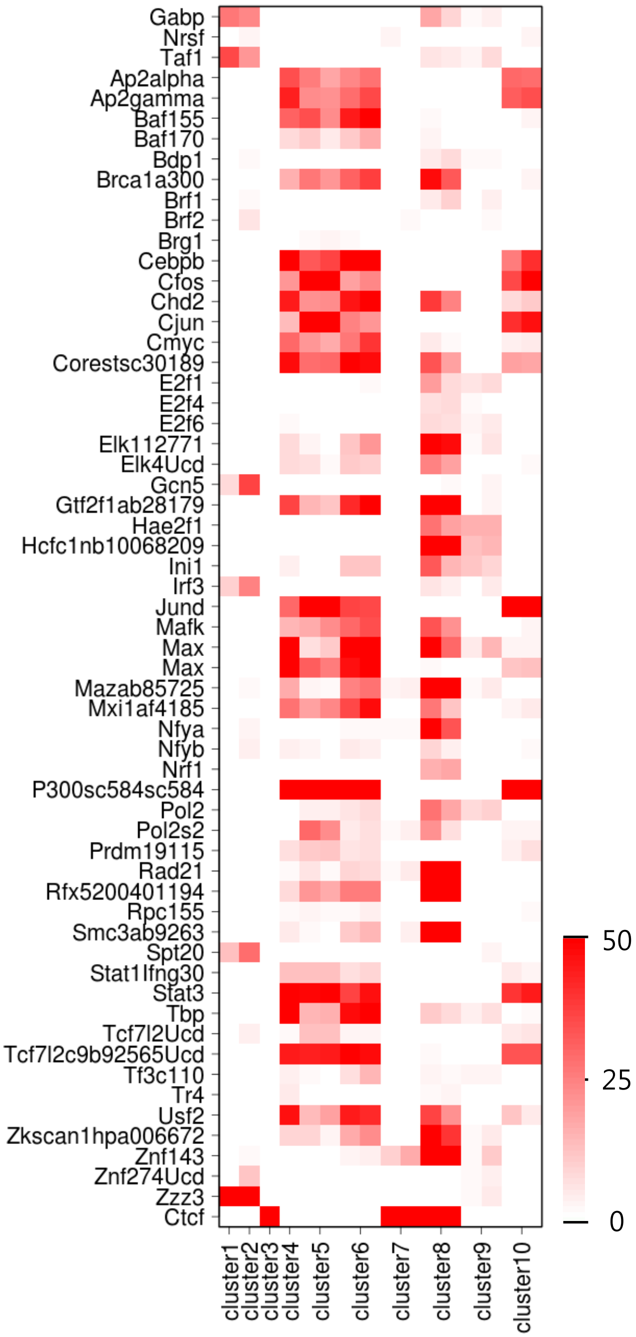

Gabp
Nrsf
Taf1
Ap2alpha
Ap2gamma
Baf155
Baf170
Bdp1
Brca1a300
Brf1
Brf2
Brg1
Cebpb
Cfos
Chd2
Cjun
Cmyc
Corestsc30189
E2f1
E2f4
E2f6
Elk112771
Elk4Ucd
Gcn5
Gtf2f1ab28179
Hae2f1
Hcfc1nb10068209
Ini1
Irf3
Jund
Mafk
Max
Max
Mazab85725
Mxi1af4185
Nfya
Nfyb
Nrf1
P300sc584sc584
Pol2
Pol2s2
Prdm19115
Rad21
Rfx5200401194
Rpc155
Smc3ab9263
Spt20
Stat1Ifng30
Stat3
Tbp
Tcf7l2Ucd
Tcf7l2c9b92565Ucd
Tf3c110
Tr4
Usf2
Zkscan1hpa006672
Znf143
Znf274Ucd
Zzz3
Ctcf
cluster1
cluster2
cluster3
cluster4
cluster5
cluster6
cluster7
cluster8
cluster9
cluster10
50
25
0

Figure S7

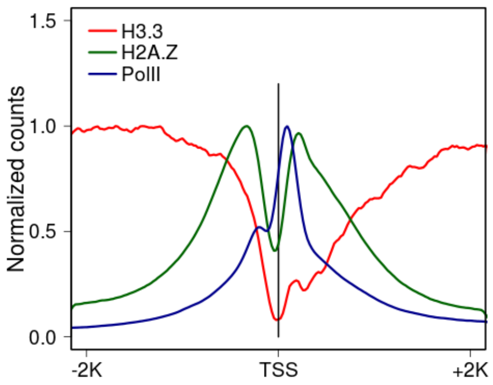

Supplement: Additional file 3: — Figure S1. The averaged profiles of histone variants in association with gene expression. Figure S2. Histone variants’ codes and their shapes. Figure S3. Histone variants and other factors. Figure S4. PolII is enriched to the direction of H3.3. Figure S5. Comparison of eNA levels and PolII levels. Figure S6. Transcription factors and co-factors enriched at each cluster significantly enriched at each cluster. Figure S7. The averaged profiles of H3.3, H2A.Z, and PolII at the TSSs of active genes. [file 13072_2015_5_MOESM3_ESM.pdf]
